# Enumeration and targeted analysis of *KRAS*, *BRAF* and *PIK3CA* mutations in CTCs captured by a label-free platform: Comparison to ctDNA and tissue in metastatic colorectal cancer

**DOI:** 10.18632/oncotarget.13350

**Published:** 2016-11-15

**Authors:** Evelyn Kidess-Sigal, Haiyan E. Liu, Melanie M. Triboulet, James Che, Vishnu C. Ramani, Brendan C. Visser, George A. Poultsides, Teri A. Longacre, Andre Marziali, Valentina Vysotskaia, Matthew Wiggin, Kyra Heirich, Violet Hanft, Ulrich Keilholz, Ingeborg Tinhofer, Jeffrey A. Norton, Mark Lee, Elodie Sollier-Christen, Stefanie S. Jeffrey

**Affiliations:** ^1^ Department of Medicine, Division of Hepatology and Gastroenterology, Charité University Hospital, Berlin, Germany; ^2^ Department of Surgery, Stanford University School of Medicine, Stanford, CA, USA; ^3^ Vortex BioSciences, Inc., Menlo Park, CA, USA; ^4^ Department of Pathology, Stanford University School of Medicine, Stanford, CA, USA; ^5^ Boreal Genomics, Vancouver, BC, Canada; ^6^ Counsyl Inc., San Francisco, CA, USA; ^7^ Comprehensive Cancer Center Charité, Berlin, Germany; ^8^ Department of Radiooncology and Radiotherapy, Charité University Hospital, Berlin, Germany; ^9^ GRAIL, Redwood City, CA, USA

**Keywords:** colorectal cancer, circulating tumor cells, circulating tumor DNA, liquid biopsy, Vortex

## Abstract

Treatment of advanced colorectal cancer (CRC) requires multimodal therapeutic approaches and need for monitoring tumor plasticity. Liquid biopsy biomarkers, including CTCs and ctDNA, hold promise for evaluating treatment response in real-time and guiding therapeutic modifications. From 15 patients with advanced CRC undergoing liver metastasectomy with curative intent, we collected 41 blood samples at different time points before and after surgery for CTC isolation and quantification using label-free Vortex technology. For mutational profiling, KRAS, BRAF, and PIK3CA hotspot mutations were analyzed in CTCs and ctDNA from 23 samples, nine matched liver metastases and three primary tumor samples. Mutational patterns were compared. 80% of patient blood samples were positive for CTCs, using a healthy baseline value as threshold (0.4 CTCs/mL), and 81.4% of captured cells were EpCAM+ CTCs. At least one mutation was detected in 78% of our blood samples. Among 23 matched CTC and ctDNA samples, we found a concordance of 78.2% for KRAS, 73.9% for BRAF and 91.3% for PIK3CA mutations. In several cases, CTCs exhibited a mutation that was not detected in ctDNA, and vice versa. Complementary assessment of both CTCs and ctDNA appears advantageous to assess dynamic tumor profiles.

## INTRODUCTION

Colorectal cancer (CRC) is the third most common cancer diagnosed worldwide in both men and women [1, 2]. Although colorectal cancer screening tests can detect cancer at an early stage, and also prevent development of colorectal cancer by removal of precancerous polyps, only around 59% of the U.S. population undergo screening [3], resulting in the diagnosis of only 39% of cancers at a localized stage [4]. Whereas 5-year relative survival is excellent for patients with localized disease (90%), it is substantially lower for patients with regional (70%) or distant (13%) metastases [5].

For CRC patients, mutation status of *KRAS* and *NRAS* in the primary tumor are currently routinely assessed after surgical resection by PCR-based methods to evaluate whether future therapeutic administration of EGFR inhibitors may be successful [6-8]. *RAS* (*KRAS* and *NRAS*) status may, however, differ between the primary tumor and metastatic lesions, due to intratumor heterogeneity and subclonal evolution [9, 10]. Nevertheless, repeat tissue biopsies are associated with risk of complications of invasive procedures, and metastatic sites may be difficult to access. Further, due to limited sampling, a tissue biopsy may only provide information on a fraction of the genomic variation within a tumor, and malignant cells may even be overlooked. Therefore, reliable markers more comprehensively representing the molecular makeup of the cancer genome in real-time would be helpful for detecting treatment resistance mechanisms and disease progression [11, 12].

Circulating tumor cells (CTCs) have been identified to be an important link between primary tumors and metastases. CTCs are cells that can detach from the tumor, enter the blood stream after undergoing a process called epithelial-to-mesenchymal transition (EMT) and survive the shear stress as well as the immune system in circulation [13, 14]. Some of these cells can then extravasate, undergoing the reverse process called mesenchymal-to-epithelial transition (MET) and initiate development of metastases at distant sites [15]. Because of their important role in the metastatic process, the number of CTCs in a cancer patient's blood may be used to assess cancer prognosis [16], to evaluate treatment response, to monitor cancer recurrence or minimal residual disease, and potentially to detect cancer in a screening setting [17]. Beyond enumeration, analysis of the proteins, DNA and RNA from CTCs can inform the development of tailored therapies for individual patients. Despite growing interest in this “liquid biopsy”, it is a challenge to detect and characterize CTCs, as they are very rare: even in patients with advanced disease, as few as a single CTC may be present in several milliliters of blood in a background of millions of leukocytes (5–10×10^6^) and billions of red blood cells (approximately 5×10^9^) [18].

Although CTCs were first described over a century ago [19, 20], deep characterization has only recently become possible with advances in CTC enrichment [21] as well as sequencing technologies. The most common enrichment platforms to date have been based on the identification of circulating cells expressing epithelial markers, most notably epithelial cell adhesion molecule (EpCAM). As an example of this label-dependent approach, the CellSearch platform has been extensively studied in the application to CTC detection in patients with CRC [22–27]. Other EpCAM-dependent methods that have been used to detect CTCs from patients with CRC include: Adna Test, which uses antibodies against EpCAM and MUC-1 conjugated to magnetic beads [28], the CTC-Chip, a microfluidic platform containing microposts coated against EpCAM [29], and magnetic-activated cell separation (MACS), another anti-EpCAM immunomagnetic enrichment method [30]. A main disadvantage of these platforms is their reliance on EpCAM expression on CTCs. Cells undergoing EMT may lack this marker and thus be missed with such an approach [31]. In this regard, multiple EpCAM-independent technologies have been used to investigate CTCs in CRC, including several filter-based enrichment platforms, which exploit the larger size of tumor cells compared to blood cells [32, 33], and a fiber-optic array scanning technology (FAST) that combines immunofluorescent staining with high-throughput imaging to enable cytomorphometric analysis of the CTCs [34].

Recently, circulating tumor DNA (ctDNA), also known as tumor-derived cell-free DNA (cfDNA), has emerged as another liquid biopsy assay, with early evidence to support its use in many of the same applications as CTCs [13, 35–39]. ctDNA is thought to be released into the blood stream by tumor cells by active secretion or as a consequence of apoptosis or necrosis, with its presence and amount generally reflecting tumor burden. ctDNA needs to be distinguished from wild-type cell-free DNA that is normally present in plasma as a result of usual cell turn-over; ctDNA is thus identified by measuring variant DNA sequences in circulation, which frequently mirror those seen in tumor tissue. The relationship of ctDNA to CTCs is under investigation at this time. Due to its relative abundance compared to CTCs, and its amenability to direct sequencing approaches, ctDNA may be better suited to analyses dependent on sensitivity. However, unlike CTCs, ctDNA is present in plasma in bulk form, and downstream analyses for cell-level biology (e.g. protein and RNA expression analyses) are not feasible. Systematic characterization of blood samples with parallel CTC and ctDNA analyses are needed to better understand their relationships and the relative (and likely complementary) information contributed by each approach.

**Figure 1:**
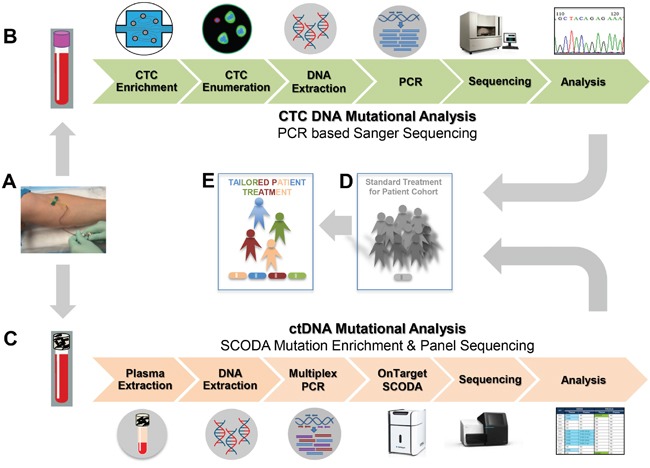
Study Workflow and aims **A.** Blood was collected from 15 patients with colorectal cancer (CRC) metastatic to the liver (2 tubes from each patient at several time points, respectively). **B.** One tube was used for CTC analysis: CTCs were enriched using the Vortex Gen1 Chip (Vortex BioSciences). Collected CTCs were fixed, stained and enumerated. Then, DNA was extracted for mutational analysis of CTC DNA by PCR based Sanger sequencing (3 genes). **C.** Another tube of whole blood was used for analysis of ctDNA: After centrifugation, plasma was collected and DNA was extracted for mutational analysis of plasma circulating tumor DNA (ctDNA). ctDNA mutational analysis was performed by panel sequencing (4 genes) after prior enrichment of targeted mutants using the SCODA mutation enrichment technology. **D.** While standard treatments are currently used for certain patient cohorts, **E.** our aim is to promote tailored cancer patient treatment.

Here, we use the Vortex Chip, a label-free microfluidic platform, to isolate CTCs from the blood of CRC patients. The Vortex Chip utilizes laminar microvortices for enrichment and downstream molecular analyses of CTCs from blood [40–42]. We compare the mutational status of CTCs to that of primary and/or metastatic tumor tissue and ctDNA as analyzed by SCODA (sequence-specific synchronous coefficient of drag alteration) mutation enrichment and detection technology [36] (Figure 1).

## RESULTS

### Analytical performance of the Vortex platform on colon cancer cell lines

At a high flow rate (4 mL/min) on the Vortex platform, cancer cells experience large inertial shear gradient lift forces and are pushed into the laminar fluid microvortices that develop in the reservoirs, where they stably recirculate. The blood cells, by virtue of being smaller, do not experience a sufficient lift force to be retained in these vortices and remain in the main flow. An additional solution exchange with a buffer injected at the same overall flow rate washes away remaining blood cells, while still maintaining the cancer cells trapped in the vortices. By lowering the flow rate, vortices dissipate, release the enriched cancer cells from the chip and allow for their capture for downstream analysis.

**Figure 2:**
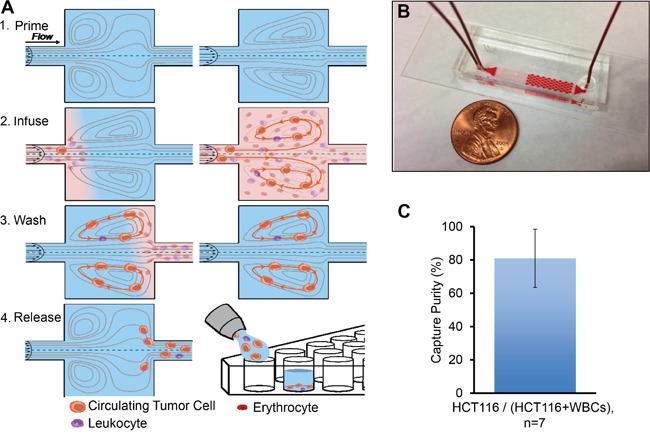
Microfluidic device design and performance **A.** Blood sample processing consists of the following steps: 1) Priming of the device with wash solution (PBS) to eliminate air bubbles. At high flow rates (4 mL/min), laminar microvortices develop in the rectangular cavities. 2) Sample Infusion: The larger cancer cells get trapped in the vortices, while smaller red blood cells (RBCs) and white blood cells (WBCs) either pass through or transiently enter vortices. 3) Wash by switching to the wash solution at the same flow rate, thereby removing RBCs and WBCs, while the CTCs remain in the vortices. 4) Release of captured cells by lowering the flow rate to dissipate the vortices. Cells are collected into wells of a 96-well plate for further downstream analysis. **B.** The device consists of molded PDMS bonded to glass and contains one wash inlet, one sample inlet, and one outlet. **C.** Capture Purity in blood is evaluated by spiking ~500 HCT116 cells into 10x-diluted blood collected from a healthy donor. After processing through Vortex device, cells were stained with DAPI, EpCAM and CD45, respectively, for visualization of the nucleus and to identify blood cells. Average capture purity (i.e. percentage of contamination with WBC) is shown for cell spiking into blood.

As an initial evaluation of platform performance for CRC cells, HCT116 cancer cell lines were spiked both into PBS and 10x diluted blood from healthy donors to characterize capture efficiency and purity based on immunostaining for EpCAM (epithelial cell-surface marker), CD45 (a leukocyte cell-surface marker), and DAPI (a nuclear stain) (Figure 2C, Supplementary Figure S1). Capture purity was 81% (Figure 2C). Capture efficiency was similar in PBS and blood (20% and 28%, respectively), which may indicate that the blood viscosity has a low influence on the capture rate (Supplementary Figure S1). Captured cells remain viable (data not shown) and may be collected in a concentrated volume of ~150 μL, which is suitable for most downstream analyses [41].

### Enumeration of CTCs from CRC patients

41 blood samples from 15 patients were collected at different time points prior to and after surgical resection of liver metastases and processed through the Vortex chip device. 10 blood samples from 10 healthy donors were collected and processed similarly. Patient information is summarized in Supplementary Table S1 and Supplementary Figure S2.

**Figure 3:**
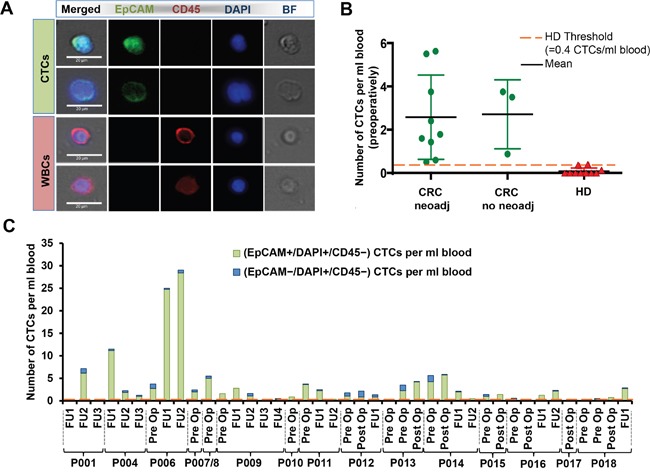
CTC immunostaining and enumeration **A.** Representative images of CTCs and WBCs stained with EpCAM, CD45, and DAPI. Scale bar represents 20 um. **B.** The number of CTCs per ml blood was determined for each patient. The number of CTCs in the preoperative blood draws are depicted by green circles for patients that received or did not receive neoadjuvant treatment. The number of CTCs in samples from age-matched healthy donors (HD) are depicted by red triangles. CTC numbers were higher in CRC patients compared to HD. A threshold was set at 0.4 CTCs per ml blood (mean HD + 2 standard deviations). **C.** The number of EpCAM^+^/DAPI^+^/CD45^-^ CTCs compared to the number of EpCAM^-^/DAPI^+^/CD45^-^ CTCs is shown for all patients at different time points (pre-, postoperatively and during follow-up visits) by green and blue stacked bars, respectively. Orange dotted lines represent the threshold of 0.4 CTCs/ml, above which positive cells are counted as CTCs.

Briefly, in addition to morphologic criteria (see Supplementary Materials), cells that stained EpCAM+/CD45-/DAPI+ or EpCAM-/CD45-/DAPI+ with both a nucleus size above 9 μm and a nucleus-to-cytoplasm (N:C) ratio above 0.6 were classified as CTCs, and cells with EpCAM-/CD45+/DAPI+ were classified as white blood cells (WBCs) (Figure 3A).“

Using the classification criteria, more cells with a CTC phenotype were found in preoperatively collected colon cancer patient samples (mean: 2.6 CTCs/mL, range: 0.5 – 5.6 CTCs/mL) than in age-matched controls (mean: 0.1 CTCs/mL, range: 0 – 0.4 CTCs/mL) (Figure 3B). We observed no significant difference in preoperative blood samples from patients who received or did not receive neoadjuvant treatment: For patients who underwent neoadjuvant treatment (P007-009, P011-012, P014-016, P018): mean 2.6 CTCs/mL, range: 0.5 – 5.6 CTCs/mL; for patients with no treatment (P006, P010, P013): mean 2.2 CTCs/mL, range 0.9 – 3.8 CTCs/mL. Considering all blood samples independently of the time of collection, 0.1 – 29 CTCs/mL (mean: 3.4 CTCs/mL) were collected in CRC patient blood samples, along with 4 – 517.4 WBCs/mL (mean: 32.5 WBCs/mL), corresponding to a capture purity of 0.3 – 63.5% (mean: 14.5%). For defining a sample as being CTC positive, a threshold was set at 0.4 CTCs per mL of blood. This healthy baseline threshold value was determined by calculating the mean number of candidate CTC-like cells found in 10 age-matched healthy donors (HD) + 2 standard deviations [0.1+2(0.15) = 0.4]. In a paper by Ozkumur E et al. [43], that also used a microfluidic technology for CTC isolation, blood from 13 healthy donors processed using their microfluidic chip showed a CTC detection cutoff at 0.5 cells per mL, similar to our threshold of 0.4 CTCs per mL. Using this baseline value as a threshold, 80% (33 of 41 samples) of CRC cancer samples processed were found to be positive for CTCs. The captured CTCs displayed varying levels of EpCAM expression (from low to high expression), with 81.4% of captured cells defined as CTCs showing an EpCAM+/DAPI+ phenotype (Figure 3C). This is in accordance to the known fact, that most CRC CTCs show a high EpCAM expression and was the reason to use this marker for visualization of CTCs in this study [44].

### Comparison of CTC levels with clinical parameters

**Figure 4:**
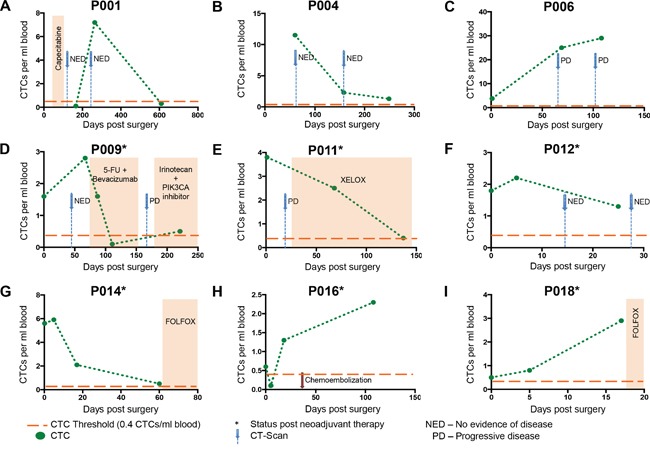
Longitudinal CTC enumeration results Graphs **A-I** showthe evolution of CTC numbers during the clinical course and treatment of a subset of CRC patients with hepatic metastases. Orange colored bars represent Chemotherapy. Black stars behind patient numbers indicate status post neoadjuvant therapy prior to resection of liver metastases. Blue arrows depict computer-tomography scans. Orange dotted lines represent the HD threshold of 0.4 CTCs/ml, above which positive cells are counted as CTCs. NED = no evidence of disease. PD = progressive disease.

P009, P011, P012, P014, P015 and P018 (Figures 4D-4I) all had received neoadjuvant therapy prior to surgical resection of liver metastases. In two out of nine cases, the number of CTCs showed consistency to clinical parameters (Figure 4C, 4E): P006 (Figure 4C) showed rapidly rising CTC numbers, which was reflected in the patient's CT-scans showing progressive disease; for P011, CTC numbers were high, and a CT-scan revealed progressive disease (Figure 4E), but after administration of chemotherapy, CTC numbers declined, revealing good response to therapy.

In three out of the nine cases, (P001, P004 and P012, Figure 4A, 4B, and 4F) CTC numbers did not mirror clinical findings: In patient P001, after surgical resection of liver metastases and postoperative chemotherapy, no CTCs were detectable, which was reflected in CT-scan results that showed ‘no evidence of disease’ (NED) (Figure 4A). However, we later observed a transient rise in CTC numbers during the third follow-up visit of this patient, around 9 months after surgery, while at the next revisit 21 months post-surgery, again no CTCs were detectable, even though no further chemotherapy had been administered. In P004 (Figure 4B), CTC numbers postoperatively declined, but remained detectable in follow-up blood draws, while imaging results showed no evidence of disease. For P012 (Figure 4F), we constantly were able to detect CTCs, whereas CT scans showed no evidence of disease.

For the remaining four cases (Figure 4D, 4G-4I), additional clinical follow-up is needed for final interpretation concerning the clinical relevance of CTC quantities: In P009, a CT scan around 40 days after surgery and prior to adjuvant chemotherapy showed no evidence of disease, although CTCs were still detectable (Figure 4D). After administration of chemotherapy to P009, CTC numbers declined, revealing good response to therapy. Following completion of the first cycle of adjuvant therapy, a CT-scan indicated progressive disease, which was accompanied by a detectable rise in CTCs. P014 showed a slow decline of CTC numbers after surgery whereas P018 showed rising numbers (Figure 4G and 4I), which may indicate minimal residual disease. Both patients received adjuvant chemotherapy with FOLFOX. P016 had hepatic metastases in both liver lobes, so that only one was resected, and resection was planned for the other after chemoembolization (Figure 4H). Interestingly, 5 days after surgery, there were no CTCs detectable, but later the numbers started to rise.

Taken together, our data show, that CTC levels parallel the clinical course for selected cases, but for others, additional clinical follow-up is needed to determine whether the CTCs detected may represent active residual tumor ultimately emerging as metastatic disease.

### Comparison of CTC levels with ctDNA and CEA levels

**Figure 5:**
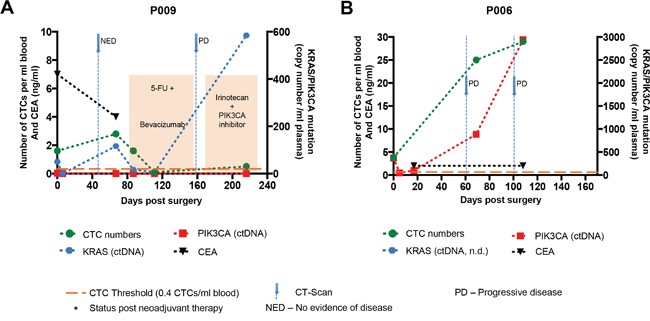
Longitudinal CTC enumeration results in comparison to ctDNA and CEA levels Graphs **A** and **B** showthe development of CTC numbers alongside ctDNA and CEA levels during the clinical course and treatment of two CRC patients with hepatic metastases. Orange colored bars represent chemotherapy. Black stars behind patient numbers indicate status post neoadjuvant therapy prior to surgical resection of liver metastases. Blue arrows depict computer-tomography scans. Orange dotted lines represent the HD threshold of 0.4 CTCs/ml, above which positive cells are counted as CTCs. NED = no evidence of disease. PD = progressive disease.

Additionally, we analyzed CTC, ctDNA and CEA levels in two patients to simultaneously assess these biomarkers over time and to determine which of these markers best reflected the completeness of surgical resection, therapeutic response and disease recurrence (Figure 5A&5B). CTC numbers and ctDNA levels showed similar dynamics in both patients. Interestingly, postoperative CEA values were normal (<5 ng/ml) whenever analyzed in both patients. In P009, who had received neoadjuvant treatment prior to surgical resection of hepatic metastases (as well as the primary tumor), CTCs and a *KRAS* and a *PIK3CA* mutation in ctDNA were detectable preoperatively. On the 5^th^ postoperative day, plasma analysis showed no evidence of ctDNA. Two months after surgery, while the *PIK3CA* mutation remained undetectable, CTCs again were detected and the *KRAS* mutation in ctDNA was detectable at higher levels compared to the preoperative samples, possibly reflecting residual disease, although CT scan results showed no evidence of disease. After initiation of adjuvant chemotherapy, both CTC numbers and the *KRAS* mutation level declined. Eventually, around day 160, the patient showed progressive disease on imaging, and concurrently rising levels of CTCs and *KRAS* mutant ctDNA despite administration of systemic therapy (Figure 5A). For P006, where high quantities of CTCs were identified in whole blood and *PIK3CA* mutants in plasma ctDNA, postoperative imaging surveillance revealed progressive disease which was accompanied by rapidly rising levels of CTCs and *PIK3CA* mutant DNA, with up to 29 CTCs per mL blood collected at the last time point. These cases illustrate that CTCs as well as ctDNA can potentially reveal disease recurrence as well as disease progression earlier than imaging and offer additional information beyond CEA.

### Comparison of genomic mutational profile of CTCs to hepatic metastasis, primary tumors, and ctDNA

For nine patients in the cohort, mutational analyses for *KRAS*, *BRAF*, and *PIK3CA* hotspots were available for matched tumor tissue, CTCs, and ctDNA.

We used cancer cells from cell lines of known mutational status to first verify that Sanger sequencing could detect target mutations for samples with a purity of 7.5% and above (Supplementary Figure S4). We then performed Sanger sequencing to detect mutations in CTCs, and to be consistent, in primary tumors and hepatic metastases. Analysis of ctDNA samples had been performed previously by targeted panel next generation sequencing (NGS) [36].

Of three primary tumor samples analyzed, two showed mutations and these same mutations were identified in the corresponding hepatic metastases (Table 1). Of three cases with no detectable mutations in liver metastases, two cases showed no CTC or ctDNA mutations and one case had identifiable *KRAS* and *PIK3CA* mutations in ctDNA but not in CTCs. Of the six cases with mutations in hepatic metastases, five (83%) had the same mutation identified in CTCs and five (83%) had the mutation identified in ctDNA. In case P001, a *KRAS* mutation was identified in CTCs but not ctDNA; and in case P016, a *BRAF* mutation was identified in ctDNA but not in CTCs. Overall, of 23 blood draws testing three genes for mutations in these 9 patients (69 opportunities for testing CTC and ctDNA concordance during treatment), simultaneous mutational status of *KRAS* in CTCs and ctDNA were matched in two blood draws and discordant in five, with 16 blood draws showing no mutations in CTCs or ctDNA; mutational status of *BRAF* showed a match between CTCs and ctDNA in one blood draw and discordance in six, with 16 blood draws showing no mutations in CTCs or ctDNA; and mutational status of *PIK3CA* showed a match between CTCs and ctDNA in six blood draws and discordance in two, with 15 blood draws showing no mutations. These 13 mutationally discordant assays represent 59% of the 22 assays that showed any mutations, and 19% (13/69) of all the mutational assays performed. Of the 13 discordant assays for individual genes, four detected mutations in CTCs without detectable mutations in ctDNA, and nine showed mutations in ctDNA without corresponding mutations detected in the CTCs. Of these nine blood samples that showed only ctDNA mutations, four had CTC purity less than 7.5%, bringing into question whether a mutation may have gone undetected in these CTCs using Sanger sequencing. When grouping all three genes tests together as a panel, in three patients (P006, P009, and P013), our analysis revealed the presence of two different mutations concurrently (*BRAF* and *PIK3CA* in CTCs of P006; *KRAS* and *PIK3CA* in ctDNA of P009; and *KRAS* and *PIK3CA* in CTCs and ctDNA of P013). Overall, tissue and CTC mutational profiles or tissue and ctDNA profiles both matched in 7 of 9 patients (78%). In one case, CTC analysis revealed a *BRAF* V600E mutation (P006), which was not detected in the corresponding liver metastasis, suggesting tumor heterogeneity, which would have been missed if only the hepatic metastasis had been analyzed. In another case (P009), ctDNA detected mutations that were not identified in the liver metastasis. In 10 age-matched healthy donors without a history of cancer, there were no detectable mutations (Supplementary Table S3).

**Table 1:** Mutation detection in primary tumor (FFPE), liver metastatic tissue, CTCs and plasma ctDNA in Colorectal cancer patients

Donor ID	Time Point of Collection	KRAS				BRAF				PIK3CA				CTCs/ml	Purity %
		Primary	Liver Met	CTCs	ctDNA	Primary	Liver Met	CTCs	ctDNA	Primary	Liver Met	CTCs	ctDNA		
**P001**	**Pre-Op**		**G12D**				**ND**				**ND**				
	**Follow up 1**			**ND**	**ND**			**ND**	**ND**			**ND**	**ND**	**0.1**	**0.6**
	**Follow up 2**			**G12D**	**ND**			**ND**	**ND**			**ND**	**ND**	**7.2**	**39.4**
	**Follow up 3**			**ND**	**ND**			**ND**	**ND**			**ND**	**ND**	**0.3**	**3.0**
**P006**	**Pre Op**		**ND**	**ND**	**ND**		**ND**	**V600E**	**ND**		**H1047R**	**H1047R**	**H1047R**	**3.8**	**23.8**
	**Follow up 1**			**ND**	**ND**			**ND**	**ND**			**H1047R**	**H1047R**	**25.0**	**54.6**
	**Follow up 2**			**ND**	**ND**			**ND**	**ND**			**H1047R**	**H1047R**	**29.0**	**63.5**
**P007**	**Pre Op**		**ND**	**ND**	**ND**		**ND**	**ND**	**ND**		**ND**	**ND**	**ND**	**2.4**	**14.3**
**P008**	**Pre Op**		**ND**	**ND**	**ND**		**ND**	**ND**	**ND**		**ND**	**ND**	**ND**	**5.5**	**43.4**
**P009**	**Pre Op**	**ND**	**ND**	**ND**	**G12D**	**ND**	**ND**	**ND**	**ND**	**ND**	**ND**	**ND**	**E542K**	**1.6**	**0.3**
	**Follow up 1**			**ND**	**G12D**			**ND**	**ND**			**ND**	**ND**	**2.8**	**32.0**
	**Follow up 2**			**ND**	**G12D**			**ND**	**ND**			**ND**	**ND**	**1.6**	**10.9**
	**Follow up 3**			**ND**	**G12D**			**ND**	**ND**			**ND**	**ND**	**0.1**	**0.4**
**P013**	**Pre Op**	**G13D**	**G13D**	**G13D**	**G13D**	**ND**	**ND**	**ND**	**ND**	**H1047L**	**H1047L**	**H1047L**	**H1047L**	**3.5**	**15.3**
	**Post Op**			**G13D**	**G13D**			**ND**	**ND**			**H1047L**	**H1047L**	**4.3**	**11.8**
**P014**	**Pre Op**		**ND**	**ND**	**ND**		**V600E**	**V600E**	**ND**		**ND**	**ND**	**ND**	**5.6**	**9.0**
	**Post Op**			**ND**	**ND**			**V600E**	**V600E**			**ND**	**ND**	**5.9**	**22.9**
	**Follow up 1**			**ND**	**ND**			**ND**	**ND**			**ND**	**ND**	**2.1**	**7.5**
	**Follow up 2**			**ND**	**ND**			**V600E**	**ND**			**ND**	**ND**	**0.5**	**11.1**
**P015**	**Pre-Op**		**ND**	**ND**	**ND**		**ND**	**ND**	**ND**		**E542K**	**E542K**	**E542K**	**1.4**	**5.4**
	**Post-Op**			**ND**	**ND**			**ND**	**ND**			**ND**	**E542K**	**1.4**	**7.2**
	**Pre Op**	**ND**	**ND**	**ND**	**ND**	**V600E**	**V600E**	**ND**	**V600E**	**ND**	**ND**	**ND**	**ND**	**0.6**	**9.7**
**P016**	**Post Op**			**ND**	**ND**			**ND**	**V600E**			**ND**	**ND**	**0.1**	**0.6**
	**Follow up 1**			**ND**	**ND**			**ND**	**V600E**			**ND**	**ND**	**1.3**	**19.6**

Grey indicates that no sample was available. Blue and purple represent biopsies from primary tumor (FFPE) and liver metastatic tissue, respectively. CTC samples and plasma ctDNA samples collected at different time points are represented in green and orange, respectively. ND = mutation not detected.

Overall, for each gene within a blood draw, we found a concordance of 78.2% (18/23) for *KRAS*, 73.9% (17/23) for *BRAF*, and 91.3% (21/23) for *PIK3CA*, among a total of 23 blood and plasma samples. In several cases, CTCs exhibited a mutation that was not detected in ctDNA: for example, in P001 follow-up 2, a *KRAS* G12D mutation was detected, while in P014 preoperatively and at follow-up 2, a *BRAF* V600E mutation was detected in CTCs but not in ctDNA. On the other hand, there were several cases showing exactly the opposite, namely a mutation detected in ctDNA but not in CTCs. For example, in P009, *KRAS* mutations were detected in all ctDNA samples collected but neither in the tissues nor in the CTCs, while the preoperatively collected plasma ctDNA sample harbored a concurrent *PIK3CA* mutation. In P016, *BRAF* V600E mutations were detectable at all ctDNA sample collection times, which was concordant to primary and hepatic tumor tissue analyses, but was not detectable in the CTCs. Similarly, in P015, the analysis of the postoperative blood and plasma samples only showed the *PIK3CA* mutation in plasma ctDNA. Here, it needs to be kept in mind that the total number of CTCs per mL of sample (DNA yield), as well as sample purity play important roles for detecting CTC mutations with Sanger sequencing: The number of CTCs per mL of blood was very low in P016 (DNA yield < 10pg), while sample purity was under the 10% detection limit preoperatively and at follow-up 3 in P009, and postoperatively in P015 (Table 1). This would require more sensitive techniques to enhance the limit of detection. In sum, while taking into account these limitations, our results indicate that CTCs and ctDNA are complementary and both should be evaluated in parallel to gain more detailed information on the mutational landscape of a patients' tumor.

## DISCUSSION

In the study presented here, we describe the use of a novel microfluidic device (Vortex Biosciences Inc.) for the label-free isolation of CTCs from blood of patients with surgically resectable CRC metastatic to the liver and compare mutational analyses of CTCs with ctDNA and tumor tissue. PCR-based sequencing for hot spot mutations of *KRAS*, *BRAF* and *PIK3CA* genes in CTCs revealed similar mutations as tumor biopsies for 77.8% of the patient samples. Comparison of CTC and ctDNA profiling showed similarities, but with additional mutations detected either in CTCs or ctDNA, which confirms the importance of both approaches.

The Vortex technology used here allows isolation and enrichment of CTCs with high purity. Whereas some label-free CTC isolation methods may require a red blood cell lysis (RBC lysis) step or buffy-coat separation prior to enrichment [45, 46], this is not necessary using this device, which avoids preprocessing and potential loss of rare valuable CTCs. Traditionally, CTCs have been captured by antibody-based methods targeting EpCAM, an epithelial marker not expressed on white blood cells. Interestingly, after DAPI/EpCAM/CD45 staining and enumeration of the CTCs collected, we were able to show that the majority of CTCs from our patient cohort were EpCAM-positive. Still, EpCAM-negative subclones that have undergone epithelial-to-mesenchymal transition (EMT), which are postulated to be crucial for development of metastases [31], were also captured. Next, using Vortex technology, normalized per tube of blood, up to 217.5 CTCs/7.5 mL of blood were captured (healthy cut-off at 3 CTCs/7.5 mL), with a mean capture purity of 14.5%. This represents more CTCs with higher purity compared to the CellSearch system (which is associated with poor prognosis when > 3 CTCs/7.5 mL of blood are present) [25, 43, 47].

Although the Vortex platform is capable of capturing live CTCs, in this study we were limited by blood sample volume, and so used the same CTCs that had been collected into a well-plate, then fixed and stained for enumeration, for downstream genomic analysis: using the same sample enabled the detection of both CTC numbers and CTC gene mutations by using only one portion of blood with one time processing.

PCR-based Sanger sequencing is a traditional sequencing approach that allows fast and economic DNA sequencing, and is still considered the “gold standard” for validation studies in a smaller setting [48]. Advantageously, PCR-based Sanger sequencing does not require whole-genome amplification (WGA), which added bias when performed on fixed cells, producing false negative and false positive results (data not shown). For this purpose, we optimized a complete workflow for fixed cells, including optimized DNA extraction and PCR-based Sanger sequencing. Using this workflow, we analyzed captured CTCs as pooled cells and looked for the existence of hotspot mutations in *KRAS*, *BRAF* and *PIK3CA* genes. This was possible due to the relatively high purity obtained in most CTC samples collected (mean purity 14.5%) given that Sanger sequencing detected mutations in fixed cells at a purity level of 7.5% or more (Supplementary Figure S4). In contrast, 30% (7/23) of CTC samples had a purity less than 7.5%, and mutations were not detected in 5 of these samples. Of 13 samples where no mutations were detected in CTCs, 5 (38%) had CTC purity less than 7.5% (Table 1), highlighting a concern whether or not CTC mutations existed in these fixed CTC samples, and suggesting that future studies should include a more sensitive sequencing technique for CTCs, such as NGS on fresh cells or droplet-based digital PCR [49-51].

*KRAS* and *BRAF* mutation status are predictive for whether a patient may respond to treatment with EGFR-inhibitors, such as cetuximab [7-10]. Hence, detection of these mutations is important in the clinical setting for CRC. Additionally, *NRAS* mutations are known to cause resistance to anti-EGFR agents [7-9], but in in this study we focused on the gene mutations mentioned above, with higher frequency in CRC [52]. We compared the CTC mutational analysis results to corresponding hepatic metastatic tissue and primary tissue, whenever available. Our results indicate that CTCs can be used for non-invasive monitoring of the mutational pattern, as we observed a relatively high concordance of 77.8% (7/9) between mutations found in CTCs compared to tissue biopsies. In one case, a mutation was identified in CTCs but not in tissue, which may suggest that this reflects a rare subclone in the tissue with intratumor heterogeneity. Similarly, Speicher's group has shown that mutations identified in single CTCs from the blood of patients with metastatic CRC which were not initially found in corresponding primary or metastatic tumor tissue could later be identified in the tissue using ultradeep sequencing, again suggesting a subclonal origin [53]. Such mutations may be missed if only the tumor biopsy is analyzed and provides evidence that CTCs may reflect the molecular makeup of a patients' tumor more comprehensively than a tissue biopsy. Further, CTCs can be collected repeatedly in a non-invasive manner. Moreover, others have shown that CTC gene expression in metastatic CRC may better reflect the molecular features of metastatic deposits rather than primary tumor [54].

When comparing mutations detected in CTCs and ctDNA, we found that in some blood samples, CTCs revealed a mutation that was not detected in ctDNA, while in others, ctDNA revealed mutations that weren't observable in CTCs. This suggests that CTCs and ctDNA are both needed to be evaluated in the clinical setting to enable optimal surveillance of the course of disease and treatment selection.

The CTC enrichment platform used in this study coupled with PCR-based Sanger sequencing exhibits good performance and offers added advantages of speed and simplicity in assessment of mutation in CTCs. However, we acknowledge that there are some limitations to our workflow. First, by using the Vortex platform, with a capture efficiency estimated around 27% from HCT116 cells spiking experiments, some CTCs may have been missed. A new generation of the device made of a different material and that includes a significantly larger number of trapping chambers was developed to tackle this limitation. Recent testing shows higher capture efficiency while maintaining similar capture purity. Recovery is further enhanced using multiple rounds of reprocessing, with higher cumulative efficiency; such improvements will need to be tested in a future study. Second, CTCs were analyzed by Sanger sequencing, which has much lower sensitivity than NGS, and may have missed mutations in samples with low CTC purity. Next, by analyzing hot spot mutations of 3 genes in CTCs and ctDNA, other mutations may have been missed. Also, the number of patients and matched clinical samples is limited. Thus, further studies including a considerably larger patient cohort using the same mutation analysis method on both CTC and ctDNA and on corresponding tissue samples are needed in the future to verify our interesting results and especially to more accurately compare the different signatures in CTC and ctDNA samples. Finally, blood sampling site (e.g., CTCs or ctDNA obtained centrally from a hepatic vein versus from a peripheral vein) may be an important variable to consider in future investigations of metastatic CRC [55, 56], as it has been demonstrated that a significantly higher number of CTCs can be isolated from mesenteric blood of colorectal cancer patients compared to peripheral blood, which indicates that the liver effectively filters the CTCs in those patients [56]*.*

Our study is one of a few comparing mutational analysis results from CTCs to the ones obtained from ctDNA in the same patient cohort, as well as mutational analyses on corresponding tissue specimens. Sundaresan et al. recently described the detection of a hot spot mutation that leads to an acquired resistance to tyrosine kinase inhibitors for patients with non-small cell lung cancers, with similar results in that complementary approaches are best to completely assess an individual patients' cancer [57].

Biomarkers are crucial to guide treatment decisions. In CRC, information on *KRAS*, *BRAF* and *PIK3CA* genotype is extremely valuable in systemic chemotherapy. Analysis of these three gene mutations in CTCs or ctDNA, which reflect certain subpopulations of the primary tumor as well as cells forming metastases, should be considered as an important “liquid biopsy” method to monitor the development of the disease and its status over time, and particularly for its clinical impact in guiding drug selection. At present, extracting CTCs with high recovery and purity from the high background of blood cells still represents a challenge for the CTC field, and has limited the broad use of CTCs in clinical settings. Our study demonstrates the complementary use of targeted mutational analyses of CTCs captured by a label-free platform and ctDNA for monitoring of tumor evolution.

## MATERIALS AND METHODS

### Patients and samples

Blood and tissue specimens, as well as clinical data, were collected from a total of 15 patients with stage IV colorectal cancer with surgically resectable metastatic disease to the liver. Blood specimens were also collected from 10 age-matched healthy donors without history of malignant disease. All patients were recruited at Stanford University Hospital, according to clinical study protocol approved by the ethics and Institutional Review Board (Stanford IRB # 5630).

Prior to being enrolled into the study, all patients provided written informed consent. For the cancer cases, eligible patients had adenocarcinoma of the colon or rectum with surgically resectable hepatic metastases (Stage IV disease) [58], as well as available blood samples. 10 of 15 patients (67%) received standard preoperative chemotherapy regimens prior to surgical resection of hepatic metastases (5-fluorouracil, leucovorin, and oxaliplatin (FOLFOX) or FOLFOX + bevacizumab, capecitabine and oxaliplatin (XELOX) + bevacizumab, or 5-fluorouracil, leucovorin, and irinotecan (FOLFIRI) + cetuximab). Blood samples were collected into 10 ml EDTA coated tubes (BD) for CTC isolation, and Cell-Free DNA BCT® (Streck) tubes for isolation of ctDNA. All cancer patients had blood samples collected prior to surgery. For a subset of these patients, additional blood collections were performed post-operatively and at follow-up visits. 0.5-1g of metastatic tumor tissue was collected during surgery, transported on ice and stored at -80°C until further processing. Additionally, primary tumor FFPE samples were collected from 3 patients.

### Processing of patient blood samples

Collected blood specimens were stored at room temperature and processed within 4 hours after blood was drawn. An amount of 4 to 10 ml of blood was collected into an EDTA tube and diluted 1:10 with PBS, and processed to yield CTCs collected into wells of a 96 well-plate. Optimization of whole blood dilution has been previously described [41]. The enriched cells were then fixed, stained, imaged and enumerated similarly to the cancer cell lines (see below). Enriched cells were subsequently stored at 4°C until further processing. Blood samples collected into Cell-Free DNA BCT**®** (Streck Inc., Omaha, NE) tubes were subjected to centrifugation at 3200 rpm for 15 minutes, and plasma was subsequently isolated and stored at -80°C until further analysis.

### Microfluidic Vortex device design and operation

Vortex technology is comprised of a PDMS microfluidic device which geometry has been described previously [41] (Figure 2B). Flow was driven through the device by the use of two syringe pumps (Harvard Apparatus), one for the sample solution and one for the wash solution [41]. *Priming*: The device was initially primed with PBS wash solution at 4 mL/min for 30s. *Capture:* For cell capture within the reservoirs, cell sample solution and wash solution were co-infused at 3.5 mL/min and 0.5 mL/min. *Wash:* Solution exchange was performed by bringing the wash flow rate back to 4 mL/min while stopping the sample solution flow. *Release*: By stopping the total flow, vortices can dissipate and cells were released from the vortices off the chip (Figure 2A). The captured cells were released into a 96-well plate for fixation, immunostaining, imaging and enumeration.

### Characterization of the Vortex platform with cancer cell lines

The HCT116 cancer cell line was used to characterize device performance, and cancer cell lines SW620 and M395 were used to characterize DNA workflow. Adherent cells were released with 0.25% (w/v) Trypsin (Gibco®, ThermoFisher), resuspended in media and rocked gently on a shaker 30 min prior to spiking experiments. Approximately 500 cells were spiked into either 5 mL of PBS, or 5 mL of 10x diluted blood (0.5 mL of blood and 4.5 mL of PBS) and infused through the device. Once collected, the cells were fixed, stained, imaged and enumerated. Capture purity was calculated as the number of target cells collected over the total number of captured nucleated cells (Figure 2C). Capture efficiency was calculated as the number of captured target cells over the total number of target cells injected through the device (Supplementary Figure S1).

### Cell immunostaining and enumeration

Cells isolated through Vortex were fixed in 4% PFA (paraformaldehyde) (Electron Microscopy Sciences) for 10 min, blocked with 5% goat serum (Invitrogen) for 30 min, and then immunostained with DAPI (Life Technologies), anti EpCAM-fluorescein isothiocyanate (EpCAM-FITC, clone EBA-1, BD Biosciences), and anti CD45-phycoerythrin (CD45-PE, clone HI30, BD Biosciences). Using the Axio Observer Z1 microscope (Zeiss), stitched images of stained cells were acquired, and cells were manually enumerated by two different persons following the same criteria (Supplementary Figure S3). The classification criteria used for cell identification was previously described [42]. CTCs were defined as DAPI-positive/CD45-negative, either EpCAM-positive or EpCAM-negative with both a nucleus size above 9 μm and a nucleus-to-cytoplasm (N:C) ratio above 0.6. The number of EpCAM-positive and EpCAM-negative CTCs and leukocytes were documented, and the number of cancer cells per milliliter of whole blood was calculated.

### Use of cell lines with known mutations to optimize and validate genomic workflow for CTCs

We used HCT116, SW620 and M395 cancer cells to test our mutation assays. HCT116 cells harbor heterozygous hotspot mutations of *KRAS* G13D (c.38G>A) and *PIK3CA* H1047R (c.3140A>G). SW620 cells have the homozygous *KRAS* G12V (c.35G >T) mutation. M395 cells carry the homozygous *BRAF* V600E (c.T1799>A) mutation (Supplementary Figure S4). Because CTC cell number is low, giving insufficient DNA yield for use of next generation sequencing (NGS) technology, whole genome amplification (WGA) is necessary. As our CTCs had been previously fixed for enumeration, we found that WGA on fixed cell line cells followed by NGS resulted in both lower coverage of reads and uneven amplification, which caused false negative and false positive mutations (data not shown). Thus, all the mutational analyses on our fixed CTCs (and to be consistent, on tissue samples) were performed by PCR and Sanger sequencing as described below.

To determine DNA input limits for accurate Sanger sequencing of gene mutations, a range of cancer cell line DNA from 20pg to 1 ng with known mutation status were subjected to Sanger sequencing. To determine limitations in terms of sample purity of CTCs captured along with background WBCs, DNA from cancer cell lines cells was mixed with DNA from leukocytes (WBCs) in various ratios and processed for mutation detection using Sanger sequencing.

### DNA extraction from CTCs and DNA quantification

Prior to DNA extraction from fixed CTCs, the best DNA extraction method leading to the highest yield of DNA was determined by testing different kits from different vendors with different protocols on fixed HCT116 cells (data not shown). A modified protocol using the QIAamp Micro Kit (Qiagen) was then used for the DNA extraction from CTCs collected in a well-plate, fixed with 4% PFA and immunostained. Briefly, the well plates were centrifuged and PBS was cautiously removed. Tissue lysis buffer ATL and proteinase K were added to the cell suspensions followed by an overnight incubation at 60 °C. Then, the lysate was transferred from the well plate to a microcentrifuge tube. Lysis buffer AL was added to continue the lysis step. The whole lysate was then loaded into the provided column. After being washed by adding buffers AW1 and AW2, the bound DNA was eluted in 25 μl of water. As the CTC numbers are rather low, a sensitive and accurate method is needed to quantify the DNA amount. Therefore, we performed a quantitative PCR to determine the amount of DNA by using human long interspersed nuclear element-1 (hLINE-1) primers (Forward: 5′-TCACTCAAAGCCGCTCAACTAC-3′and Reverse: 5′-TCTGCCTTCATTTCGTTATGTACC-3′). The hLINE-1 gene is ideal for this purpose, because ~100,000 of these elements exist in the human genome [59].

### DNA extraction from tissue specimens and DNA quantification

DNA from 10 mg of frozen metastatic liver tissues was extracted by using the QIAamp DNA Micro Kit following the tissue protocol (Qiagen). DNA from 10 μm of FFPE samples of primary cancer tissues (5μm per section, 2 sections) was extracted by using the GeneRead DNA FFPE Kit (Qiagen). The extracted DNA was quantified by Qubit Fluorometer (ThermoFisher Scientific). 1 ng of purified DNA was then subjected to PCR directly.

### PCR and nested PCR

The extracted DNA was subjected to PCR in a total volume of 40 μl containing 20 μl of AmpliTaq Gold® 360 PCR Master Mix (Life Technologies), 2 μl of corresponding primers (10 μmol/L) and 5 μl of extracted CTC DNA or 1 ng of tissue DNA as the template. Primers of the genes *KRAS*, *BRAF* and *PIK3CA* were designed to produce amplicons covering hotspot regions of *KRAS* codons 12 and 13, *BRAF* V600E, as well as *PIK3CA* codons 542, 545 (exon 9) and 1047 (exon 20) (Supplementary Table S2) and were synthesized by Elim Biopharmaceuticals, Inc.

Thermal cycler conditions were: 95°C for 5 min, 35 cycles of 94°C for 30 sec, 58°C for 45 sec, 72°C for 45 sec and finally 10 min at 72°C for the final extension. For *PIK3CA* exon 9, a nested PCR was done by using the nested primers and 2 μl of PCR product from the first PCR reaction with the following conditions: 95°C for 5 min, 25 cycles of 94°C for 30 sec, 58°C for 45 sec, 72°C for 45 sec and finally 10 min at 72°C for the final extension. All PCR experiments were done inside the AC600 PCR Workstation (AirClean Systems) with cautious handling to eliminate any contamination. The PCR products were checked by E-Gel Electrophoresis System (ThermoFisher Scientific) and measured by the Qubit. Remaining PCR products were then purified using the QIAquick PCR Purification Kit (Qiagen). The purified PCR product was measured by Qubit before being sent out for sequencing.

### Sanger sequencing

Purified PCR product was sent to Elim Biopharmaceuticals for Sanger sequencing. All reactions were run on a 3730XL DNA Analyzer (ThermoFisher Scientific) and sequenced by using the M13 forward primers. The Sanger sequence ABI chromatogram files were analyzed by using the BioEdit sequence alignment editor.

### Analysis of ctDNA

Analysis of ctDNA mutation profiles using the multiplexed synchronous coefficient of drag alteration (SCODA) mutation enrichment and detection platform were performed as previously described [36]. Briefly, DNA was extracted from collected plasma samples, and after addition of internal positive controls (IPCs), the DNA samples were subjected to a multiplex PCR reaction, using primers with sample-specific barcodes. Amplified samples were then enriched for mutant DNA (46 mutations in the four genes *KRAS*, *BRAF*, *PIK3CA* and *EGFR*) using the multiplexed SCODA mutation enrichment technology. The library was then constructed from the enriched mutant DNA, quantified by quantitative PCR and sequenced on the MiSeq platform (Illumina).

## SUPPLEMENTARY MATERIALS DATA


