# Supplementary material for: Enumeration and targeted analysis of KRAS, BRAF and PIK3CA mutations in CTCs captured by a label-free platform: Comparison to ctDNA and tissue in metastatic colorectal cancer
Source: Oncotarget. 2016 Nov 15;7(51):85349–64. doi: 10.18632/oncotarget.13350 (PMC5356741; doi:10.18632/oncotarget.13350)
Supplement: Supplementary file 1 [file oncotarget-07-85349-s001.pdf]

Enumeration and targeted analysis of *KRAS*, *BRAF* and *PIK3CA* mutations in CTCs captured by a label-free platform: Comparison to ctDNA and tissue in metastatic colorectal cancer

SUPPLEMENTARY DATA

Cell culture

Colon cancer cell lines HCT116 and SW620 were purchased from the American Type Culture Collection (ATCC). M395 were derived from tumor biopsies of metastatic melanoma lesions progressing after an initial objective response to vemurafenib treatment (donation from Dr. R. Kulkarni, UCLA Dermatology). All cells were grown at 37°C and 5% CO2 in Gibco® RPMI 1640 medium (HCT116, M395) or Leibovitz's L-15 Medium (SW620), supplemented with 10% fetal bovine serum and 1% Penicillin-Streptomycin.

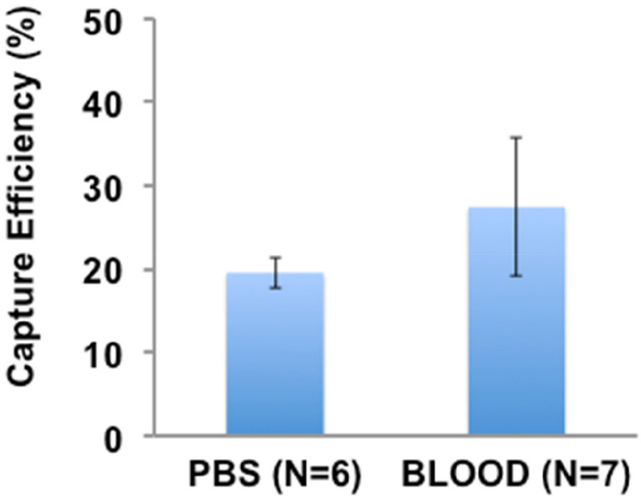

Supplementary Figure S1: Capture Efficiency in PBS vs. Blood. Spiking of ~500 HCT116 cells into 5 mL PBS or 5 mL of 10x-diluted blood from a healthy donor, respectively. Sample was processed through Vortex Gen1 device at a flow rate of 4 ml/min [41]. Cells were collected into wells of a 96-well plate and stained with DAPI for visualization of the nucleus, as well as with EpCAM and CD45 in samples spiked into blood, to be able to identify white blood cells. Cells with a positive DAPI signal after spiking into PBS, or cells positive for EpCAM and DAPI, but negative for CD45 after blood spiking, respectively, were counted as HCT116 cells. Average capture efficiency is depicted by blue bars for HCT116 cell spiking into PBS vs. blood, respectively. Error bars represent the standard deviation obtained for N independent experiments.

A

No.	Age	Sex
HD1	62	F
HD2	41	F
HD3	69	M
HD4	46	M
HD5	45	M
HD6	55	M
HD7	77	M
HD8	30	M
HD9	51	F
HD10	80	F

B

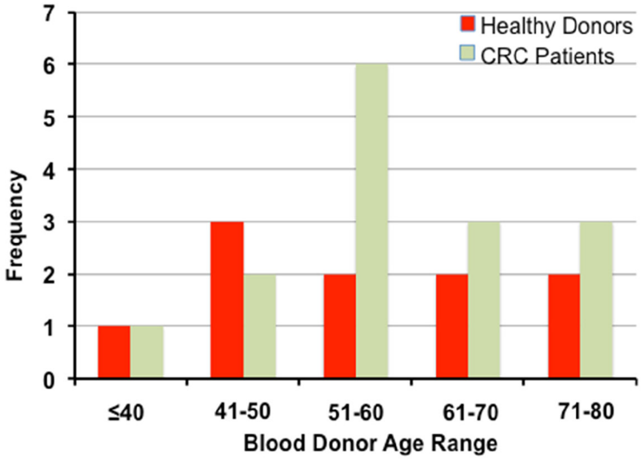

Supplementary Figure S2: A. Healthy Donor characteristics. **B.** Age distribution for Healthy donors (n=10) vs. CRC patients (n=15).

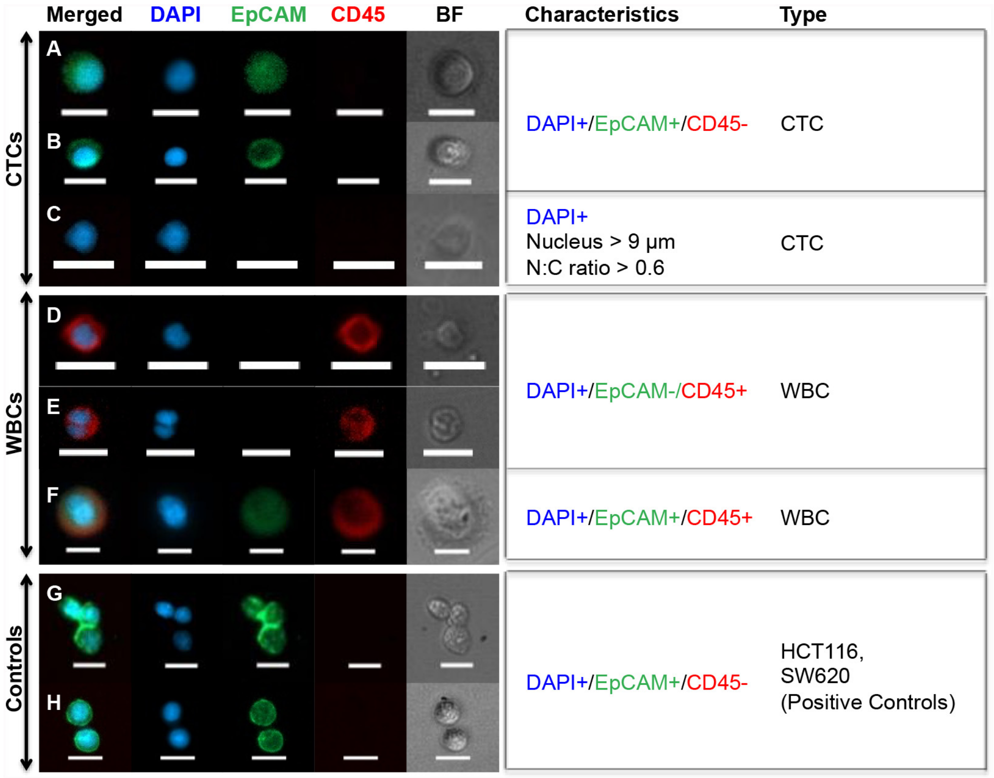

Supplementary Figure S3: Gallery of cells collected from CRC patient blood samples after DAPI, EpCAM, CD45 immunostaining and morphology-based cell classification. A, B. DAPI+/CD45-/EpCAM+ circulating tumor cells (CTCs). **C.** DAPI+/CD45-/EpCAM- CTCs. CTCs are defined as DAPI+/CD45-, either EpCAM+ or EpCAM- with both a nucleus size above 9 µm and a nucleus:cytoplasmic (N:C) ratio above 0.6. **D, E.** DAPI+/CD45+/EpCAM- white blood cells (WBCs). **F.** DAPI+/CD45+/EpCAM+ WBCs. A cell which stains DAPI+/EpCAM-/CD45+ or double-stains EpCAM+/CD45+ is counted as a WBC. Our enumeration criteria also defines cells DAPI+ only with a nucleus size below 9 µm, or above 9 µm AND N:C ratio below 0.6 as a WBC. **G, H.** HCT116 and SW620 colorectal cancer cell lines are used as positive controls for EpCAM staining for each patient sample.

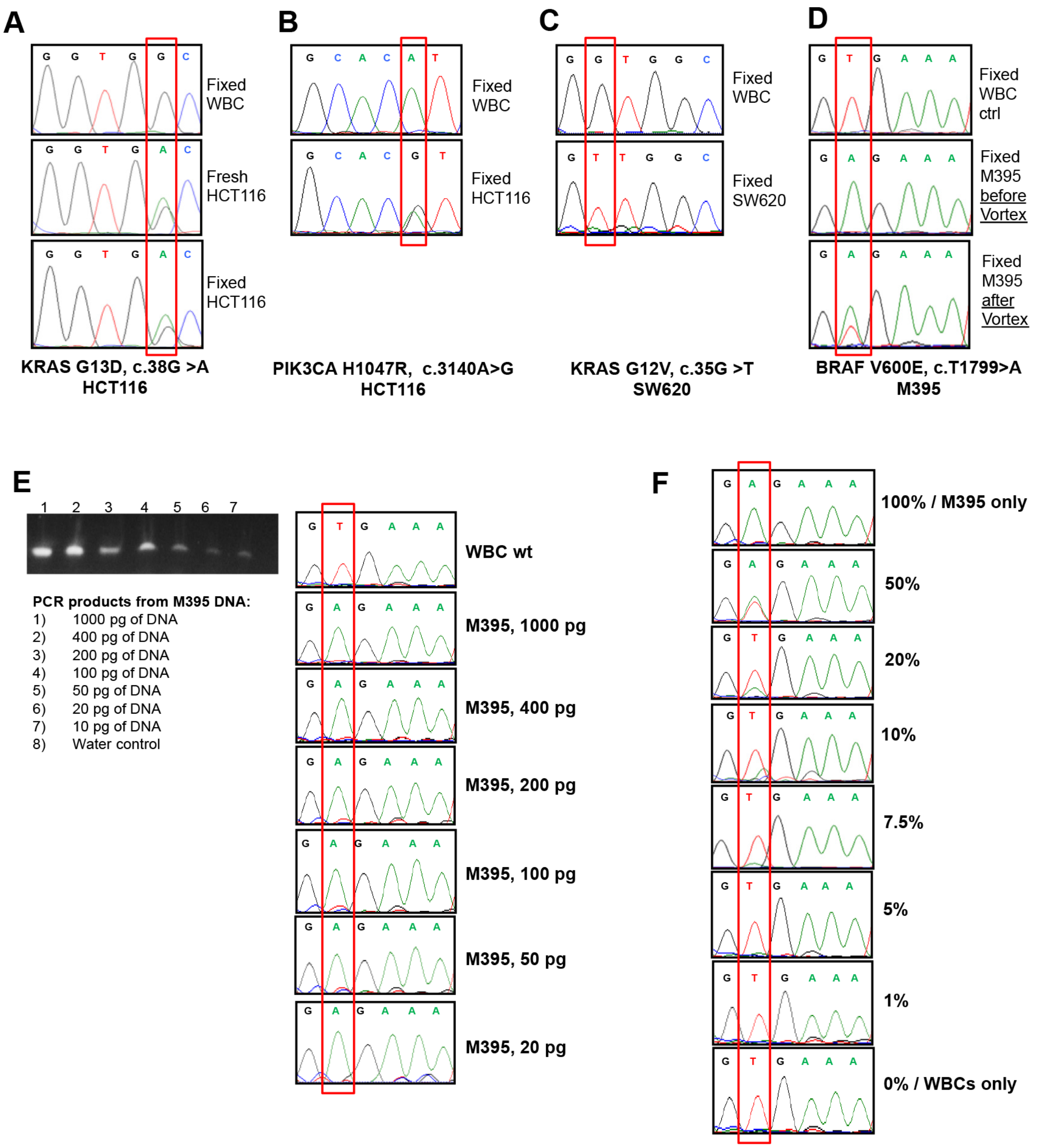

Supplementary Figure S4: Primers and workflow verification. A. *KRAS* G13D heterozygous mutation was confirmed on both fixed and fresh HCT116 cells. Cell fixation did not prevent mutation detection. **B.** *PIK3CA* heterozygous mutation was confirmed on fixed HCT116 cells. **C.** *KRAS* G12V homozygous mutation was confirmed on SW620 cells. **D.** *BRAF* V600E homozygous mutation was detected on M395 cells, before and after spiking into blood and processing through Vortex device. **E.** Analysis of DNA input limits showed that even with a DNA yield less than 20pg, PCR still gives accurate sequencing results. **F.** Using different ratios of HCT116 DNA with white blood cell (WBC) DNA as background, Sanger sequencing workflow demonstrated an accurate detection of gene mutations for sample purities as low as 7.5-10%.

Supplementary Table S1: Patient characteristics

No.	Age	Sex	TNM, Grade	Preoperative Chemotherapy	Liver metastases	Margin status	Postoperative Chemotherapy
P001	66	M	T2 N0 M1, G1	No	solitary	R0	Yes
P004	59	M	T3 N0 M1, G2-3	No	multiple	R0	No
P006	39	M	T3 N1 M1, G3	No	multiple	R1	No
P007	58	M	T3 N2a M1, G2	Yes	multiple	R0	Yes
P008	52	M	T3 N1 M1, G2	Yes	multiple	R0	Yes
P009	57	F	T4 N2 M1, G2	Yes	multiple	R0	Yes
P010	42	F	T3 N1 M1, G2	No	solitary	R0	Yes
P011	72	M	T3 N2 M1, G2	Yes	solitary	R0	Yes
P012	47	F	T3 N1 M1, G2	Yes	multiple	R0	No
P013	68	M	T3 N1 M1, G1	No	multiple	R0	No
P014	64	M	T4 N1 M1, G2	Yes	multiple	R0	Yes
P015	50	F	T4 N2 M1, G2	Yes	multiple	R1	No
P016	71	F	T3 N2 M1, G2	Yes	multiple	R0 (1st step of two step operation for resection of liver metastases)	Portal vein embolization
P017	70	M	T2 N0 M1, G2	Yes	multiple	R0	No
P018	51	F	T3 N2 M1, G2	Yes	multiple	R0	Yes

Resection margin status R0=No residual tumor microscopically; R1=microscopically residual tumor [58].

Supplementary Table S2: Primers

Gene Name	Region	Primer name	Sequences	Amplicon(bp)
KRAS	Exon 2, Codon 12 & 13	KRAS-exon2-S-For-M13	5′- tgtaaaacgacggccagt TGTGACATGTTCTAATATAGTCACATT-3‘ [a]	237
		KRAS-exon2-S-Rev-M13	5′- caggaaacagctatgacc ACCAGTAATATGCATATTAAAACAAGA-3′ [a]	
BRAF	Exon 15, Codon 600	BRAF-exon15-For-M13	5'-tgtaaaacgacggccagt CTAAACTCTTCATAATGCTTGCTC-3‘ [a]	293
		BRAF-exon15-Rev -M13	5'-caggaaacagctatgacc TCTAGTAACTCAGCAGCATCTCA-3‘ [a]	
PIK3CA	Exon 9, Codon 542, 543, 544, 545, 546, 547, 548	PIK3CA-e9-For-M13	5′- tgtaaaacgacggccagt GTAACAGACTAGCTAGAGAC-3‘ [b]	211
		PIK3CA-e9-Rev-M13	5′- caggaaacagctatgacc ACATGCTGAGATCAGCCAAA-3′	
		PIK3CA-e9-Rev-M13-Small	5′-caggaaacagctatgacc AGCACTTACCTGTGACTCCA-3′	151
	Exon 20 Condon H1047, H1048	PIK3CA-e20-For-M13	5′- tgtaaaacgacggccagt CGAAAGACCCTAGCCTTAGA-3'	181
		PIK3CA-e20-Rev-M13	5′-caggaaacagctatgacc TTTTCAGTTCAATGCATGCTG-3′[b]	

Sequences of the primers used for the mutation detection of *KRAS* exon 2, *BRAF* exon 15, *PIK3CA* exon 9 and *PIK3CA* exon 20. Each primer includes the M13 tail to facilitate subsequent sequencing using universal M13 primers (sequences of the M13 primers are: M13 forward 5' TGTAAAACGACGGCCAGT 3', M13 reverse 5' CAGGAAACAGCTATGACC 3‘).

[a] Applied Biosystems: application note: KRAS Variant Identification. https://tools.thermofisher.com/content/sfs/brochures/cms_072026.pdf

[b] Karapetis CS, Jonker D, Daneshmand M, Hanson JE, O'Callaghan CJ, Marginean C, Zalcberg JR, Simes J, Moore MJ, Tebbutt NC, Price TJ, Shapiro JD, Pavlakis N, Gibbs P, Van Hazel GA, Lee U, Haq R, Virk S, Tu D, Lorimer IA. NCIC Clinical Trials Group and the Australasian Gastro-Intestinal Trials Group. PIK3CA, BRAF, and PTEN status and benefit from cetuximab in the treatment of advanced colorectal cancer--results from NCIC CTG/AGITG CO.17. Clin Cancer Res. 2014 Feb;20(3):744-53.

Supplementary Table S3: Mutation detection in age-matched healthy donors (without history of a malignancy)

Donor ID	KRAS		BRAF		PIK3CA	
	CTC	ctDNA	CTC	ctDNA	CTC	ctDNA
HD1	ND	ND	ND	ND	ND	ND
HD2	ND	ND	ND	ND	ND	ND
HD3	ND	ND	ND	ND	ND	ND
HD4	ND	ND	ND	ND	ND	ND
HD5	ND	ND	ND	ND	ND	ND
HD6	ND	ND	ND	ND	ND	ND
HD7	ND	ND	ND	ND	ND	ND
HD8	ND	ND	ND	ND	ND	ND
HD9	ND	ND	ND	ND	ND	ND
HD10	ND	ND	ND	ND	ND	ND

Neither *KRAS*, *BRAF*, nor *PIK3CA* mutations were detected in age-matched donors. ND = mutation not detected.
